# Sources of nitrous oxide emissions from hydroponic tomato cultivation: Evidence from stable isotope analyses

**DOI:** 10.3389/fmicb.2022.1080847

**Published:** 2023-01-04

**Authors:** Stefan Karlowsky, Caroline Buchen-Tschiskale, Luca Odasso, Dietmar Schwarz, Reinhard Well

**Affiliations:** ^1^Leibniz Institute of Vegetable and Ornamental Crops (IGZ) e.V., Großbeeren, Germany; ^2^Thünen Institute of Climate-Smart Agriculture, Federal Research Institute for Rural Areas, Forestry and Fisheries, Braunschweig, Germany; ^3^Operation Mercy, Amman, Jordan

**Keywords:** glasshouse vegetable production, horticulture, greenhouse gas emission, N_2_O isotopocules, ^15^N labeling, denitrification

## Abstract

**Introduction:**

Hydroponic vegetable cultivation is characterized by high intensity and frequent nitrogen fertilizer application, which is related to greenhouse gas emissions, especially in the form of nitrous oxide (N_2_O). So far, there is little knowledge about the sources of N_2_O emissions from hydroponic systems, with the few studies indicating that denitrification could play a major role.

**Methods:**

Here, we use evidence from an experiment with tomato plants (*Solanum lycopersicum*) grown in a hydroponic greenhouse setup to further shed light into the process of N_2_O production based on the N_2_O isotopocule method and the ^15^N tracing approach. Gas samples from the headspace of rock wool substrate were collected prior to and after ^15^N labeling at two occasions using the closed chamber method and analyzed by gas chromatography and stable isotope ratio mass spectrometry.

**Results:**

The isotopocule analyses revealed that either heterotrophic bacterial denitrification (bD) or nitrifier denitrification (nD) was the major source of N_2_O emissions, when a typical nutrient solution with a low ammonium concentration (1–6 mg L^−1^) was applied. Furthermore, the isotopic shift in ^15^N site preference and in δ^18^O values indicated that approximately 80–90% of the N_2_O produced were already reduced to N_2_ by denitrifiers inside the rock wool substrate. Despite higher concentrations of ammonium present during the ^15^N labeling (30–60 mg L^−1^), results from the ^15^N tracing approach showed that N_2_O mainly originated from bD. Both, ^15^N label supplied in the form of ammonium and ^15^N label supplied in the form of nitrate, increased the ^15^N enrichment of N_2_O. This pointed to the contribution of other processes than bD. Nitrification activity was indicated by the conversion of small amounts of ^15^N-labeled ammonium into nitrate.

**Discussion/Conclusion:**

Comparing the results from N_2_O isotopocule analyses and the ^15^N tracing approach, likely a combination of bD, nD, and coupled nitrification and denitrification (cND) was responsible for the vast part of N_2_O emissions observed in this study. Overall, our findings help to better understand the processes underlying N_2_O and N_2_ emissions from hydroponic tomato cultivation, and thereby facilitate the development of targeted N_2_O mitigation measures.

## 1. Introduction

Based on a variety of technical innovations in greenhouse vegetable production, the use of soilless culture systems (commonly referred to as “hydroponics”) has grown in importance during the last 30–40 years (Gruda, 2009; Savvas et al., 2013; Savvas and Gruda, 2018). Controlled environment systems are considered by some as key part of future food production (Lakhiar et al., 2018; Cowan et al., 2022). This is largely due to the possibility of operating hydroponic systems in greenhouses in regions with unfavorable climatic conditions and in urban areas (Sharma et al., 2018; Small et al., 2019). Closed hydroponic systems also allow the re-utilization of drained nutrient solution from the root zone by recirculating the collected drain after mixing with stock solution. The high water and nutrient efficiency of closed hydroponic systems as well as the reduction of soil-borne diseases are considered as major advantages compared to soil-based cultivation (Gruda, 2009; Savvas and Gruda, 2018). Besides, the high water and nutrient efficiency makes hydroponic systems also interesting for the production of supplemental fresh food during space missions (Wheeler, 2017). Nonetheless, there are still losses occurring in the form of gaseous nitrogen (N) emissions, which may sum up to more than 10% of the N applied in the nutrient solution (Daum and Schenk, 1996a). Due to the high N application rate and dosage frequency in hydroponics, there is also a high potential for gaseous N emissions, in particular nitrous oxide (N_2_O) from microbial processes such as nitrification (Ni) and heterotrophic bacterial denitrification (bD; Daum and Schenk, 1996b; Lin et al., 2022). If bD is complete, N losses in the form of molecular nitrogen (N_2_) due to N_2_O reduction might also occur. So far, only a few studies investigated volatile N losses from hydroponic systems. Some of these studies found N_2_O emission factors higher than the IPCC estimate of 1% N_2_O-N for applied N fertilizer in soil cultivation (Daum and Schenk, 1996a; Hashida et al., 2014; Yoshihara et al., 2016), while others found lower N_2_O emission factors (Llorach-Massana et al., 2017; Halbert-Howard et al., 2021; Karlowsky et al., 2021).

The specialty of hydroponic systems is that inert substrates such as sand, perlite, or rock wool can be used, which limits the availability of organic carbon for heterotrophic denitrifiers. In this case, the hydroponic growing medium consists only of the substrate matrix and the supplied nutrient solution, which is mostly composed of mineral fertilizers dissolved in water. Nevertheless, bD has been considered as the main source of gaseous N emissions from hydroponic systems with inert substrates (Daum and Schenk, 1996a, 1996b, 1998). Whereas a more recent study by Lin et al. (2022) with tomato plants cultivated on peat and coir substrates found also significant shares of N_2_O produced by Ni, which depended on the substrate used. In hydroponic systems with inert growing media, various factors may favor bD over Ni activity, i.e., (i) frequent irrigation pulses, (ii) slightly acidic pH values (pH 5–6.5) in the nutrient solution, (iii) often high nitrate (NO_3_^−^) to ammonium (NH_4_^+^) ratios, and (iv) the presence of root exudates and debris. Yet, there is little knowledge on the processes underlying gaseous N emissions from hydroponic systems. In particular, it is unclear to which extend other processes such as fungal denitrification (fD), nitrifier denitrification (nD), or coupled nitrification and denitrification (cND) play a role in hydroponic systems. A study of functional microbial genes by Hashida et al. (2014) found 3–5 times higher gene copy numbers for denitrifiers than for nitrifiers, but the abundance of functional Ni and bD genes had no clear relationship with measured N_2_O emissions. N_2_ emissions from bD, which are more difficult to analyze due to the high atmospheric concentration of N_2_, have only been researched by Daum and Schenk (1996a, 1996b, 1997, 1998) in hydroponic systems, using the acetylene inhibition method. However, today, it is known that this method is not suitable to quantify N_2_ production, mainly due to catalytic decomposition of NO in presence of O_2_ (Felber et al., 2012; Nadeem et al., 2013), which cannot be excluded in the setup used in the Daum and Schenk studies (*ibid.*).

Alternative methods for detecting N_2_ emissions include (i) the use of closed chambers filled with other inert gases such as helium and the analysis of N_2_ in gas samples on a gas chromatograph (helium incubation method) (Scholefield et al., 1997), (ii) the labeling with ^15^N supplied by the fertilizer and the measurement of ^15^N contents in N_2_O and N_2_ (^15^N tracing approach) (e.g., Stevens and Laughlin, 1998; Buchen et al., 2016), and (iii) the analysis of the isotopic composition (δ^18^O, δ^15^Nbulk value and the intramolecular distribution of ^15^N in N_2_O) of the four most abundant N_2_O isotopocules, which are indicative for N_2_O production pathways, but also altered during the N_2_O reduction process (N_2_O isotopocule method) (e.g., Decock and Six, 2013; Lewicka-Szczebak et al., 2017). Unfortunately, the helium incubation method to directly measure N_2_ emissions requires a high technical effort and is very prone to leakage and is therefore mainly used for the analysis of soil cores in the laboratory (Groffman et al., 2006). Both, the N_2_O isotopocule method and the ^15^N tracing approach, require little technical effort in the field or greenhouse, can be combined with the usual chamber-based gas flux measurements for detecting N_2_O emission rates, and are suitable to assess the microbial processes that drive the N_2_O emission (Lewicka-Szczebak et al., 2020). The N_2_ isotopocule method works well with natural abundance stable isotope ratios and only requires the capacity for stable isotope analyses. However, due to the multitude of possible N_2_O processes (Butterbach-Bahl et al., 2013) and the variability found in isotope contents and fractionation factors, uncertainties of its results have to be taken into account (Wu et al., 2019). The ^15^N tracing approach allows to quantify the conversion of ^15^N-enriched substrates such as NO_3_^−^ or NH_4_^+^ to different products, including N_2_O and N_2_ (^15^N mass balance). Though to obtain sufficient ^15^N enrichment of N_2_ for detection of N_2_ production, high amounts of expensive ^15^N tracer have to be applied, limiting the use of the ^15^N tracing approach for detecting N_2_ fluxes by the experimental budget. Moreover, under ambient atmosphere, its sensitivity is quite low (Zaman et al., 2021).

In this study, we used a combination of the N_2_O isotopocule method and the ^15^N tracing approach to further shed light into the processes underlying gaseous N emissions from hydroponic systems. Analyzing the N_2_O isotopocules and using the dual isotope plot (“isotopocule mapping approach”) is the most common interpretation strategy to estimate the fractions of N_2_O produced by bD and/or nD, fD, and Ni (e.g., Lewicka-Szczebak et al., 2017). The results from N_2_O isotopocule analysis were also recently found to be in good accordance with the analysis of functional nitrifier and denitrifier genes (Lin et al., 2022). In contrast to the isotopocule method, the ^15^N tracing approach allows to estimate the fraction of N_2_O derived from bD, without overlapping nD (e.g., Deppe et al., 2017). Hence, by combining the N_2_O isotopocule method and the ^15^N tracing approach, it is possible to assess potential contributions of not well-studied microbial processes such as nD or cND in N_2_O formation. Furthermore, we used two types of ^15^N label, i.e., ^15^NH_4_^+^ and ^15^NO_3_^−^, to determine the contribution of each N form in the emitted N_2_O and to gain additional insights into N transformation processes. In our study, we focused on rock wool hydroponics and used tomato plants as a model, as the use of rock wool substrate is widespread in modern production greenhouses (Dannehl et al., 2015; Savvas and Gruda, 2018) and tomato is the most important vegetable crop worldwide (Schwarz et al., 2014). We conducted two sampling campaigns: (i) at the beginning of flowering and (ii) during fruit ripening, at which we expected different N_2_O emission rates. In previous studies with rock wool substrate, higher N_2_O emissions were found during tomato fruit ripening compared to earlier plant stages (Hashida et al., 2014; Karlowsky et al., 2021), and were attributed to shifts in plant physiology.

Overall, our aim was to better understand which microbial processes contribute to N_2_O emission from hydroponic systems to enable tailored mitigation measures. We hypothesized that bD is the main source of N_2_O emissions from hydroponic tomato cultivation on rock wool, and that NO_3_^−^ is contributing to a higher share to N_2_O emissions than NH_4_^+^. Furthermore, we assumed that most of the applied ^15^N tracer can be recovered in the labeled nutrient solution, plant biomass, and gaseous N emissions in a hydroponic system with inert rock wool substrate.

## 2. Materials and methods

### 2.1. Experimental setup and hydroponic tomato cultivation

The experiment took place in an experimental glasshouse consisting of multiple heated cabins, each with a size of 64 m^2^ and a roof top height of 4 m. Two of these cabins were used for this study, cabin no. 7 for pre-cultivating tomato plants (*Solanum lycopersicum* cv. ‘Cheramy F1’) and cabin no. 5 for conducting the experiment. Temperature in the cabins was set to 20/18°C (day/night), and roof top ventilation was opened at temperatures above 23/20°C (day/night). Shading was done automatically at photosynthetically active radiation (PAR) values above 900 μmol m^−2^ s^−1^ and artificial lighting was applied between 5:00 and 12:00 CET, if PAR values were below 180 μmol m^−2^ s^−1^. Air temperature and humidity in the cabins as well as roof top PAR were continuously monitored by a climate computer (Supplementary Figure S1). Tomato plants were sown on 26th July 2021 and after germination in moistened sand, 64 seedlings were transplanted into pre-weighed rock wool cubes (10 × 10 × 6.5 cm; Grodan B.V., Roermond, Netherlands) for further cultivation. On 2nd September each two planted rock wool cubes were put on one rock wool slab (100 × 20 × 7.5 cm; Grodan Vital, Grodan B.V., Roermond, Netherlands) at a distance of 50 cm. One-half of the planted rock wool slabs were installed in eight hydroponic units with elevated gutters in cabin no. 5, which included separate fertigation systems and were later used for the ^15^N labeling. The other half was further cultivated in cabin no. 7 in four gutters on the ground, which shared one fertigation system. In both cases, the collected drain solution (i.e., leachate) was re-used and mixed with fresh nutrient solution in storage tanks as needed (closed hydroponic system with re-circulating nutrient solution). The nutrient solution from the storage tanks was supplied to plants *via* pumps, PE tubes, and drippers inserted into the rock wool cubes. The tomato plants were supplied with a custom-made nutrient solution modified after the recipe of de Kreij et al. (2003), which had a high NO_3_^−^ to NH_4_^+^ ratio (~20:1) that was found optimal for tomato cultivation. Macro and micro nutrients were dissolved in de-ionized water targeting a pH of 5.6 and an electrical conductivity (EC) of 2 mS cm^−1^. The pH and EC values in the storage tanks were regularly monitored (Supplementary Figure S2). Tomato seedlings were supplied with an N concentration of 361 mg L^−1^ at the beginning (starter solution; 338 mg L^−1^ NO_3_^−^-N and 23 mg L^−1^ NH_4_^+^-N). After the development of the 5th truss and the first green fruits on, from 4th October, the N concentration in the nutrient solution was reduced to 165 mg L^−1^ (refill solution; 151 mg L^−1^ NO_3_^−^-N and 14 mg L^−1^ NH_4_^+^-N). The composition of the different nutrient solutions used in this study can be found in Supplementary Table S1. Each hydroponic unit in cabin no. 5 consisted of a 4 m gutter in which three rock wool slabs, two with plants and one unplanted, were placed and a nutrient solution storage tank filled up to approximately 40 L (Supplementary Figure S3). Two sampling periods were selected according to expected differences in plant N uptake and associated assimilate distribution in the root-shoot system, representing high growth and N uptake rates during early development and a more balanced assimilate distribution during fruit ripening. The first sampling and ^15^N labeling campaign were performed on 22nd and 23rd September, when the tomato plants developed the 3rd truss and first flowers. Subsequently, the 16 planted rock wool slabs (32 plants) in cabin no. 5 were completely removed (destructive sampling, described below) and replaced by the other 16 planted rock wool slabs pre-cultivated in cabin no. 7 on 24th September. The eight unplanted rock wool slabs were also exchanged with fresh rock wool slabs. To avoid carryover of ^15^N label, the hydroponic gutters were covered with plastic film below the rock wool slabs until 23rd September to reduce contact with the ^15^N-enriched nutrient solution. Both, the gutters and pumps for nutrient solution, were thoroughly cleaned with a detergent/disinfectant (MENNO Florades^®^, MENNO CHEMIE-VERTRIEB GMBH, Langer Kamp, Germany) before installing the unlabeled plants and rock wool slabs. Furthermore, the storage tanks and the tubing as well as the drippers for nutrient solution were completely replaced with new material. To ensure the supply of further growing plants with water and nutrients, larger storage tanks were used (Supplementary Figure S4) and filled up to approximately 200 l. The experiment ended with the second sampling and ^15^N labeling campaign on 3rd and 4th November, when the tomato plants developed the 8th truss and the first fruits were ripe.

### 2.2. Gas flux measurements

For measuring the gas fluxes, the closed chamber method as described by Karlowsky et al. (2021) was used. Acrylic glass chambers with two small openings for plant stems were fitted around the rock wool slabs (planted and unplanted) and sealed with foam rubber to obtain a closed headspace with a volume of approximately 16 l (Supplementary Figure S5). Over a period of 1 hour after closing, four gas samples (each 30 ml) were taken in 20 min intervals with a 30 ml syringe through a sampling port on top of the chamber. The gas samples were transferred to 20 ml glass vials with silicone/PTFE septa (type N17, MACHEREY-NAGEL GmbH & Co. KG, Düren, Germany) for transport and were analyzed on the same day by a gas chromatograph (GC 2010 Plus, Shimadzu Corporation, Kyoto, Japan) equipped with an electron capture detector (ECD) for N_2_O. The measured concentrations in μmol mol^−1^ were converted to μmol m^−3^ by applying the ideal gas law, including a correction for the temperature at the time of sampling. Afterward, gas fluxes were calculated using the R package “gasfluxes” [version 0.4–4; (Fuss et al., 2020)] by robust linear regression (except one case with only 3 time points, for which standard linear regression had to be used). Input variables used were gas concentration (μmol m^−3^), chamber volume (m^3^), time after closing the chamber (h), and area covered (m^2^). The latter was set to 1 m^2^ assuming a typical density of greenhouse-cultivated tomato plants of 2 plants m^−2^. The resulting gas fluxes in μmol m^−2^ h^−1^ were further converted to g ha^−1^ d^−1^ based on molar masses.

### 2.3. Sampling and ^15^N labeling

Natural abundance samples were taken on 22nd September and 3rd November shortly before the ^15^N labeling from each hydroponic unit in cabin no. 5 (from here on called “experimental unit”). These included plant samples, nutrient solution samples, and gas samples from planted rock wool slabs. For the latter, 140 ml of air was collected from the headspace of rock wool substrate with a syringe at the end of gas flux measurements after 1 h of N_2_O enrichment in the closed chambers. The gas samples were transferred into 120 ml crimp-cap glass vials closed with gray butyl septa (type ND20, IVA Analysentechnik GmbH & Co. KG, Meerbusch, Germany) for later stable isotope analysis. To determine natural abundance δ^15^N values of plants, the tips (first three leaflets) of 2–3 fully developed leaves from one plant in each experimental unit were sampled and dried at 80°C for at least 48 h. Approximately 15 ml of nutrient solution (mixture with leachates) was sampled from the storage tank of each experimental unit and then stored at −20°C for later δ^15^N analyses. In addition, three samples of de-ionized water were taken to determine the natural abundance δ^18^O values of the nutrient solution water.

On both dates, the ^15^N labeling took place directly after the natural abundance sampling at approximately 12:00 pm CET. The remaining nutrient solution in the experimental units was removed as far as possible and 15 l of ^15^N-labeled nutrient solution was added in the storage tanks of each unit. In a randomized way, four units received a nutrient solution with ^15^N-enriched NH_4_^+^ (^15^NH_4_^+^) and four units received a nutrient solution with ^15^N-enriched NO_3_^−^ (^15^NO_3_^−^). This was done by adding ammonium nitrate (NH_4_NO_3_; SIGMA-ALDRICH, Saint Louis, MO, United States) with 10.5/11 atom-% ^15^N (^15^NH_4_^+^/^15^NO_3_^−^) as only N source. The composition of the nutrient solution used for the ^15^N labeling can also be found in Supplementary Table S1. In total, 115 mg of ^15^N was applied to each ^15^NH_4_^+^ unit and 120 mg of ^15^N to each ^15^NO_3_^−^ unit (3.1 g NH_4_NO_3_ per unit), yielding an N concentration of 146 mg L^−1^ (comparable to the standard refill solution). To distribute the ^15^N label in the hydroponic system, drip fertigation was run continuously for 30 min after adding the ^15^N labeled nutrient solution to the experimental units. After 4 h, a first sampling to determine the ^15^N enrichment in plant, nutrient solution and gas samples took place. The sampling was done analogously to the natural abundance sampling, including the determination of gas flux rates and the collection of gas samples for isotopic analyses as well as leaf and nutrient solution samples. Following the same scheme, the last sampling took place 24 h after the labeling. This time, also samples from the tomato stems, roots and fruits were taken. From the middle of the tomato plant *ca.*, 10 cm of the stem was cut. Around 0.5 g of fresh roots was sampled from the interface of rock wool cubes and rock wool slabs, where a dense root net allowed to obtain root material without rock wool fibers. Root samples were washed in de-ionized water and dried with lint-free cellulose wipes to remove the ^15^N label from adhering nutrient solution. During the second sampling campaign, each three green fruits from different positions (top, mid, and bottom) of one plant per experimental unit were sampled. All plant samples were dried for a minimum of 48 h at 80°C before later processing for analysis. Different plants were used for obtaining plant material before labeling, 4 h after labeling, and 24 h after labeling in order to minimize sampling effects on ^15^N uptake. Gas samplings for stable isotope analysis always took place on the rock wool slab in the middle of each experimental unit, from which plant samples were taken only after the last gas sampling (24 h after labeling). On the unplanted rock wool slabs, additional gas flux measurements took place shortly before the 24 h sampling to determine the N_2_O emission potential from re-circulated nutrient solution with leachate and therein contained organic carbon.

### 2.4. Analyses on nutrient solution, plant, and gas samples

The concentrations of NO_3_^−^ and NH_4_^+^ [mg N L^−1^] were determined using flow injection analysis with photometric detection (FIAmodula; MLE GmbH, Dresden, Germany). Measurements of δ^18^O values in water samples were done by TC/EA coupled to a Delta V plus IRMS (Thermo Finnigan, Bremen, Germany) *via* a ConFlo IV interface. The δ^15^N values of NH_4_^+^ and NO_3_^−^ were determined according to Dyckmans et al. (2021) using a sample preparation unit for inorganic nitrogen (SPIN) coupled to a membrane inlet isotope ratio mass spectrometer (MIRMS; Delta plus; Thermo Finnigan) *via* a ConFlo III interface. Additional nutrient solution samples taken one day after the labeling were analyzed for their dissolved organic carbon content (DOC) using a liquiTOC analyzer (Elementar Analysensysteme GmbH, Langenselbold, Germany). Dried plant samples were transferred into 20 ml HDPE vials (Zinsser Analytic GmbH, Eschborn, Germany) and ground to a fine powder using a steel ball mill (MM400; RETSCH GmbH, Haan, Germany). Plant samples were analyzed for total N content (N_t_) and their δ^15^N values using an Elemental Analyzer (EA) Flash 2000 (Thermo Fisher Scientific, Bremen, Germany), coupled with a Delta V isotope ratio mass spectrometer *via* a ConFlo IV interface (Thermo Fisher Scientific, Bremen, Germany). Data were normalized to the international scale for atmospheric nitrogen, by analysis of the international standards USGS40 and USGS41 (L-glutamic acid). Gas samples were analyzed for N_2_O isotopocules (δ^15^N_N2O_, δ^18^O_N2O_) using a Delta V Isotope ratio mass spectrometer (Thermo Scientific, Bremen, Germany), coupled to an automatic preparation system with Precon plus Trace GC Isolink (Thermo Scientific, Bremen, Germany). In this setup, N_2_O was pre-concentrated, separated, and purified, and afterward m/z 44, 45, and 46 of the intact N_2_O^+^ ions as well as m/z 30 and 31 of NO^+^ fragment ions were determined (Lewicka-Szczebak et al., 2014). All measured delta values (δ) were expressed in permil (‰) deviation from the ^15^N/^14^N and ^18^O/^16^O ratios of the international reference standards (i.e., atmospheric N_2_ and Vienna Standard Mean Ocean Water (VSMOW), respectively).

### 2.5. Data processing and calculations

Data from the analysis of natural abundance gas samples were evaluated for δ^15^Nα (δ^15^N of the central N position of the N_2_O molecule), δ^15^Nβ (δ^15^N of the peripheral N position of the N_2_O), and δ^18^O according to Toyoda and Yoshida (1999) and Röckmann et al. (2003). The ^15^N site preference (δ^15^N^SP^) was defined as the difference of δ^15^Nα and δ^15^Nβ. The δ^18^O values of N_2_O depend on δ^18^O values of precursors, i.e., for denitrification to >80% on H_2_O-O of the nutrient solution (Lewicka-Szczebak et al., 2016). Therefore, δ^18^O values of the emitted N_2_O (δ^18^O_N2O_) were corrected for the δ^18^O values measured in the de-ionized water (δ^18^O_H2O_) and expressed as δ^18^O_N2O/H2O_ values:


(1) $$\delta^{18}O_{N2O/H2O}=\delta^{18}O_{N2O}-\delta^{18}O_{H2O}$$

In the case of nitrification, the δ^18^O_N2O_ values depend on atmospheric oxygen (O_2_) as a precursor (Kool et al., 2007). In contrast to bulk δ^15^N_N2O_, δ^15^N^SP^ is known to be independent from source processes. During chamber air sampling, the collected N_2_O was a mixture of atmospheric and substrate-emitted N_2_O. Thus, δ values of substrate-emitted N_2_O were corrected using a basic isotope mixing model according to Well et al. (2006). To calculate the contribution of N_2_O production pathways and N_2_O reduction to N_2_, the isotopocule mapping approach based on δ^15^N^SP^_N2O_ and δ^18^O_N2O_ values was applied (Lewicka-Szczebak et al., 2017; Buchen et al., 2018). For the mapping approach, literature values for δ^18^O and δ^15^N^SP^_N2O_ of bD, fD, nD, and Ni were used as proposed by Yu et al. (2020) and Lewicka-Szczebak et al. (2020). To account for differences in oxygen precursors between denitrification and Ni, the literature values for δ^18^O_N2O_ of bD, fD, and nD were adjusted by the addition of δ^18^O_H2O_ (Lewicka-Szczebak et al., 2020). Based on the sample position in the map, the contribution of bD and/or nD, Ni, and fD was calculated based on mixing equations, while the contribution of N_2_O reduction to N_2_ was calculated from the Rayleigh equation. All calculations were done as described in detail by Buchen et al. (2018) and Zaman et al. (2021) (Chapter 7: “Isotopic Techniques to Measure N_2_O, N_2_ and Their Sources). Two possible cases of N_2_O mixing and reduction were assumed: (i) N_2_O, which is produced by bD is first partially reduced to N_2_, followed by mixing of the residual N_2_O with N_2_O from other pathways or (ii) N_2_O produced by various pathways is first mixed and then reduced to N_2_. A detailed description is given in the supplement of Wu et al. (2019). Five samples from sampling 1 and four samples from sampling 2 with a low fraction of substrate-derived N_2_O were excluded from the data analyses because the uncertainty in substrate-derived δ values increases exponentially as sample and atmospheric N_2_O concentrations converge. Similar to Buchen et al. (2018), a threshold was used for the minimum difference between sample and atmospheric N_2_O concentrations, which was determined based on measured N_2_O concentrations in ambient air during the sampling. For sampling 1, the threshold was 337 ppb and for sampling 2, it was 359 ppb (65 ppb above the ambient air N_2_O concentration). This was supported by a Gaussian error propagation, with the threshold limiting the propagated errors of δ^15^N^SP^_N2O_ and δ^18^O_N2O_ to <6‰ and < 5‰, respectively.

Data from the analysis of ^15^N-enriched gas samples were only evaluated for bulk δ^15^N_N2O_. For further calculations, δ^15^N values were converted to atom-%_15N_ to express the ^15^N enrichment:


(2) $$\text{atom-}{\%}_{{\;}^{15}N}=\frac{100\%}{\frac{1}{\left(\frac{\delta {\;}^{15}N}{1000‰}+1\right)\times R_{STD}}+1}$$

with *R_STD_* being the isotopic ratio (^15^N/^14^N = 0.0036765) of atmospheric nitrogen. Calculations of the contributions of N_2_O originating from the labeled and non-labeled pools were based on the non-equilibrium distribution of N_2_O isotopocules, as described by Spott et al. (2006) and Bergsma et al. (2001). For labeling with ^15^NO_3_^−^, this approach directly determines the ^15^N enrichment of the labeled N pool producing N_2_O (ap_N2O_) and the fraction of N_2_O derived from that pool. Considering, the fraction of atmospheric N_2_O in the samples, the fraction of NO_3_^−^-derived N_2_O in the emitted N_2_O (f_PN2O_) can be calculated. A detailed procedure is given in Deppe et al. (2017). However, due to the experimental setup, labeled N_2_O could originate from two pools (NO_3_^−^, NH_4_^+^, or a mixture of both pools). Thus, for labeling with ^15^NH_4_^+^, f_PN2O_ was estimated based on the ^15^N atom fraction of emitted N_2_O (^15^a_N2O_) using a mixing equation:


(3) $$f_{PN2O}=\frac{{\;}^{15}a\;{\;}_{N2O}-{\;}^{15}aNH_{4}^{+}}{{\;}^{15}aNO_{3}^{-}-{\;}^{15}aNH_{4}^{+}}$$

with *^15^aNO_3_^−^* being the ^15^N enrichment of the NO_3_^−^ pool and *^15^aNH_4_^+^* being the ^15^N enrichment of the NH_4_^+^ pool (*cf.* Eq. 2). The N_2_O flux from the NO_3_^−^ pool (NO_3_^−^-derived N_2_O) was calculated from f_PN2O_ by ordinary linear regression using the measured N_2_O concentrations at t0 and after 1 h of chamber closure to determine the total N_2_O flux (total N_2_O), assuming that the increase in the N_2_O emitted from the ^15^N-labeled pool was also linear as shown for the emission of total N_2_O (Buchen et al., 2016). The N_2_O flux from the NH_4_^+^ pool (NH_4_^+^-derived N_2_O) was calculated analogously based on the fraction of NH_4_^+^-derived N_2_O in the emitted N_2_O (f_NH4_), which was deduced from f_PN2O_ (f_NH4_ = 1 – f_PN2O_). Thus, the NH_4_^+^-derived N_2_O was calculated as the difference between total N_2_O and NO_3_^−^-derived N_2_O.

### 2.6. Calculation of excess ^15^N and ^15^N mass balance

To determine the amount of ^15^N tracer, which was recovered in the different pools 4 and 24 h after the labeling (excess ^15^N), atom-%_15N_ values were used to calculate atom-% ^15^N excess (APE):


(4) $$APE=\text{atom-}{\%}_{{\;}^{15}N,\text{labeled}}-\text{atom-}{\%}_{{\;}^{15}N,\text{natural abundanc}e}$$

with *atom-%_15N,labeled_* being the atom-%_15N_ values of labeled samples and *atom-%_15N,natural abundance_* being the atom-%_15N_ values of natural abundance samples. Afterward, excess ^15^N [mg ^15^N unit^−1^] for each pool was calculated:


(5) $$\text{excess}\;{\;}^{15}N=\frac{APE}{100\%}\times N_{\text{pool}}$$

with *N_pool_* being the N amount in each pool [mg N unit^−1^] at the time of sampling (4/24 h after labeling). The *N_pool_* values for plant biomass were calculated by multiplying the measured dry weight [g] of shoots (leaves + stems), roots and fruits per unit with their N_t_ content [g N g_dry weight_^−1^]. The *N_pool_* values for NO_3_^−^-N and NH_4_^+^-N from the nutrient solution were calculated by multiplying the measured N concentrations [mg N L^−1^] with the total volume of nutrient solution per unit [L]. The latter was a mixture of nutrient solution added for the labeling and remaining (unlabeled) nutrient solution in the rock wool substrate. The total volume of the nutrient solution was estimated based on the dilution of NH_4_^+^-N concentrations from the labeled nutrient solution (73 mg N L^−1^ in 15 l) at the 4 h sampling point, assuming that NH_4_^+^-N concentrations in the unlabeled nutrient solutions were negligible (measured concentrations in natural abundance samples <2.5 mg N L^−1^ at first sampling campaign and <7 mg N L^−1^ at second sampling campaign) and that the N_t_ content as well as composition in the mixed nutrient solution did not substantially change during the 4 h. For the calculation of excess ^15^N, two neighboring units were excluded from the second sampling campaign, because of a spillover of labeled nutrient solution between these units. The *N_pool_* values for N_2_O were calculated from the measured gas flux rates [mg N h^−1^] of planted and unplanted rock wool slabs. For the planted rock wool slabs, cumulative N_2_O emissions [mg N] were calculated by linear integration between the natural abundance (0 h), 4 h, and 24 h samplings, and summation of hourly gas fluxes. For unplanted rock wool slabs, constant N_2_O emission rates were assumed and used to calculate cumulative N_2_O emissions, as they were not affected by plant activity. For calculating the *N_pool_* value per unit, cumulative N_2_O emissions from planted rock wool slabs were multiplied by 2 (two planted slabs per unit) and the cumulative N_2_O emissions from unplanted slabs (one per unit) were added. Finally, the excess ^15^N values from the different pools were summed up to obtain the total amount of ^15^N recovered from the labeling (^15^N_total_) and the ^15^N recovery rate [%] was calculated:


(6) $${\;}^{15}N\;\text{recovery rate}=\frac{{\;}^{15}N{\;}_{\text{total}}}{{\;}^{15}N{\;}_{\text{label}}}\times 100\%$$

with *^15^N_label_* being the amount of ^15^N tracer [mg ^15^N unit^−1^] added during the labeling.

### 2.7. Statistical analyses

All statistical analyses were done using the R software (version 4.2.0). Linear mixed-effects models were done using the R package ‘lme4’ (version 1.1–29), including the effects of individual hydroponic units as random intercept. *Post-hoc* tests on linear mixed-effects models were done using the R package “emmeans” (version 1.7.4–1), applying the Holm-Bonferroni correction method for multiple comparisons. If necessary, data were log- or square root-transformed prior to analysis to fulfill the requirements of normality and variance homogeneity.

## 3. Results

### 3.1. N_2_O flux, isotopocule, and ^15^N tracer analyses

The N_2_O flux measurements from this study are summarized in Table 1. In general, all fluxes were in the same range, except for the measurement 24 h after labeling during the first sampling, which was significantly (*p* < 0.05) higher than the other measurements. There was no significant difference between planted and unplanted rock wool slabs from the same sampling campaign. The trend to higher N_2_O emissions from unplanted substrate during sampling 2 was reflected by higher DOC contents in the nutrient solution compared to sampling 1 (Table 1).

**Table 1:** N_2_O fluxes (determined by gas chromatography) and dissolved organic carbon (DOC) concentrations at the two sampling campaigns (sampling 1, S1; sampling 2, S2).

Date	Sampling, sample	N_2_O flux (g-N ha^−1^ d^−1^)	DOC (mg L^−1^)
2021-09-22	S1, T0	0.21 ± 0.22^a^	–
	S1, T4	0.44 ± 0.27^ab^	–
2021-09-23	S1, unplanted	0.52 ± 0.55^ab^	8.9 ± 0.6^a^
	S1, T24	2.59 ± 1.32^c^	–
2021-11-03	S2, T0	0.38 ± 0.30^ab^	–
	S2, T4	0.29 ± 0.13^ab^	–
2021-11-04	S2, unplanted	0.91 ± 0.76^b^	16.8 ± 0.9^b^
	S2, T24	0.27 ± 0.16^ab^	–

^a–c^Small letters indicate significant differences (*p* < 0.05) between individual gas flux/DOC measurements. N_2_O fluxes from planted rock wool slabs were measured before ^15^N labeling (T0), 4 h after ^15^N labeling (T4), and 24 h (T24) after ^15^N labeling. N_2_O fluxes from unplanted rock wool slabs (unplanted) and DOC concentrations were measured once during each sampling campaign. Shown are average values ±SD of *n* = 8 replicates (including low N_2_O fluxes removed for stable isotope analysis of natural abundance samples).

**Figure 1:**
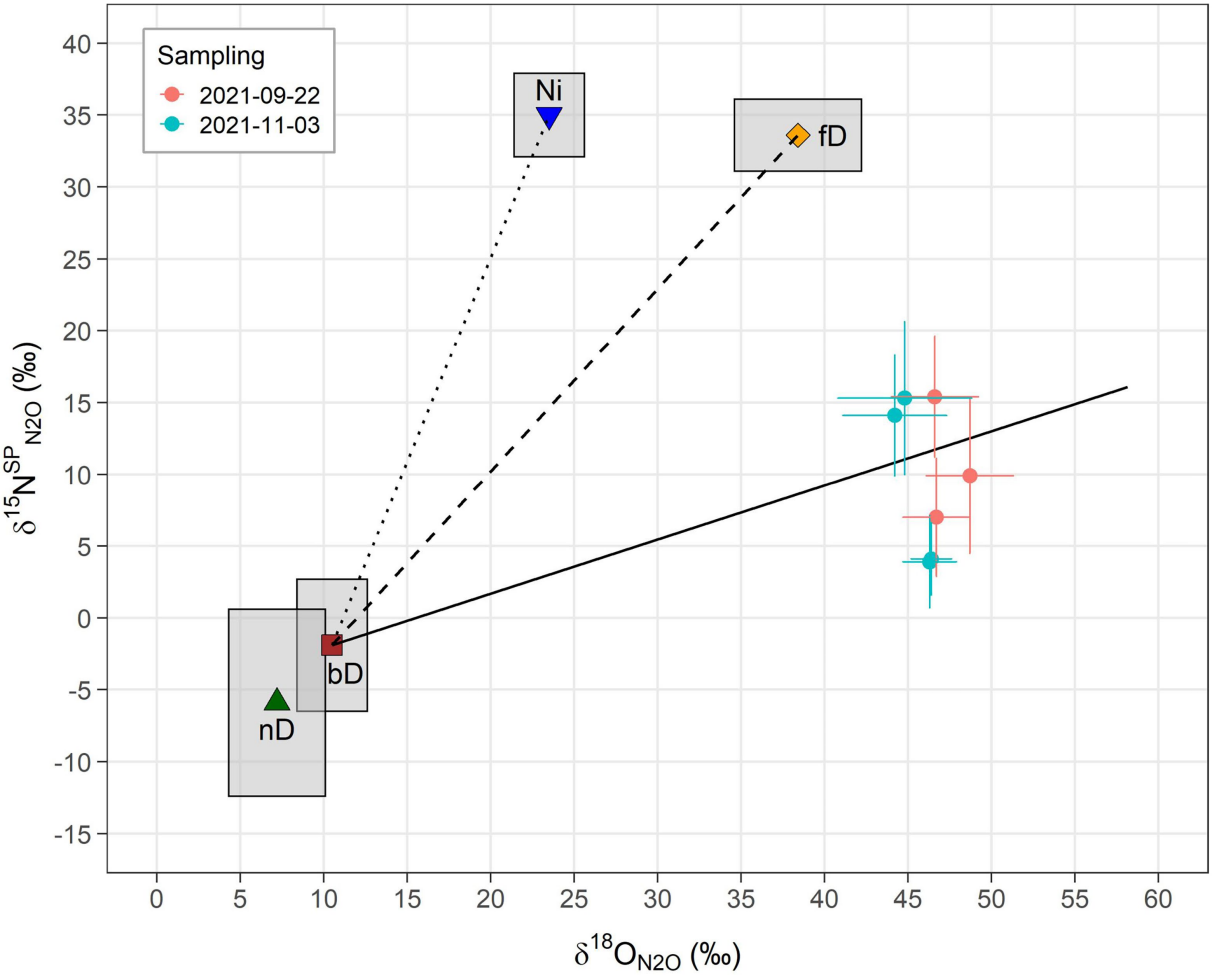
Results from N_2_O isotopocule analysis of natural abundance ^15^N gas samples illustrated as δ^15^N^SP^_N2O_/δ^18^O_N2O_ map. The vertical axis shows the ^15^N site preference of N_2_O (δ^15^N^SP^_N2O_) and the horizontal axis the abundance of the ^18^O isotope in the N_2_O molecules (δ^18^O_N2O_). Sample δ^18^O_N2O_ values were corrected for the ^18^O composition of water from the nutrient solution (δ^18^O_N2O/H2O_) as described in Eq. 1. Closed circles represent the measurement-derived values and the corresponding error bars the estimated uncertainty. Other symbols indicate literature values as compiled in Lewicka-Szczebak et al. (2020) for N_2_O produced from different microbial processes and the surrounding boxes reflect their variation (based on SD): Ni, nitrification (Yoshida, 1988; Sutka et al., 2006; Mandernack et al., 2009; Frame and Casciotti, 2010); fD, fungal denitrification (Sutka et al., 2008; Rohe et al., 2014; Maeda et al., 2015; Rohe et al., 2017); nD, nitrifier denitrification (Sutka et al., 2006; Frame and Casciotti, 2010); and bD, bacterial denitrification (Barford et al., 1999; Toyoda et al., 2005; Sutka et al., 2006; Lewicka-Szczebak et al., 2014, 2016; Rohe et al., 2017). According to Lewicka-Szczebak et al. (2020), the literature values of bD, fD and nD were adjusted by addition of the δ^18^O of water (−8.5‰) measured in this study to display expected endmember ranges. The solid line indicates the isotopic shift of N_2_O due to fractionation from the partial reduction of N_2_O to N_2_ by bD (Menyailo and Hungate, 2006; Ostrom et al., 2007; Jinuntuya-Nortman et al., 2008; Well and Flessa, 2009; Lewicka-Szczebak et al., 2014, 2015) and is shown for theoretical r_N2O_ values of 1 to 0.05. The dotted and the dashed lines represent expected values for different mixing ratios of N_2_O from bD and fD (bD-fD line) and N_2_O from bD and Ni (bD-Ni line), respectively.

Results from isotopic analyses of N_2_O are shown in Figure 1 as a δ^15^N^SP^_N2O_/δ^18^O_N2O_ map. The δ values from both samplings clearly scatter around the reduction line of N_2_O derived from bD, indicating that either bD or nD or a mixture of both was the main source of N_2_O. Moreover, the increased δ^15^N^SP^_N2O_ and δ^18^O_N2O_ values compared to the literature value for bD indicate that a high share of N_2_O was reduced before emitted to the atmosphere. Altogether, the differences in isotopic results between the first and the second sampling campaign were negligible (Table 2). Depending on which scenario (mixing of bD and fD or bD and Ni) and case (first reduction than mixing or first mixing than reduction) was assumed, the fraction of bD varied between 0.85 and 0.90, while the N_2_O/(N_2_O + N_2_) ratio of bD (r_N2O_) varied between 0.08 and 0.14. In consequence, the calculated N_2_ fluxes were between six to ten times higher than the measured N_2_O fluxes.

**Table 2:** Measured N_2_O flux, estimated fraction of N_2_O from bacterial denitrification (f_bD_), estimated N_2_O/(N_2_O + N_2_) ratio of denitrification (r_N2O_), and estimated N_2_ flux for different mixing scenarios (bacterial denitrification and fungal denitrification, bD-fD; bacterial denitrification and nitrification, bD-Ni) and cases (reduction of N_2_O from denitrification followed by mixing with N_2_O from other sources, red-mix; mixing of N_2_O from denitrification and other source followed by N_2_O reduction, mix-red).

Variable	Scenario	Case	Value sampling 1	Value sampling 2	Unit
f_bD_	bD-fD	All	0.85 ± 0.05	0.87 ± 0.13	-
	bD-Ni	All	0.88 ± 0.04	0.90 ± 0.10	
r_N2O_	bD-fD	Red-mix	0.09 ± 0.01	0.10 ± <0.01	
		Mix-red	0.13 ± 0.02	0.14 ± 0.04	
	bD-Ni	Red-mix	0.08 ± 0.01	0.09 ± 0.01	
		Mix-red	0.11 ± 0.01	0.12 ± 0.02	
N_2_O flux	All	All	1.7 ± 0.2	2.5 ± 1.0	μg N m^−2^ h^−1^
N_2_ flux	bD-fD	Red-mix	14.5 ± 0.2	19.9 ± 10.2	
		Mix-red	11.4 ± 1.0	17.8 ± 11.7	
	bD-Ni	Red-mix	17.0 ± 1.0	21.9 ± 8.8	
		Mix-red	13.8 ± 0.2	19.6±10.4	

Shown are average values ± SD (*n* = 3 for Sampling 1; *n* = 4 for Sampling 2).

**Figure 2:**
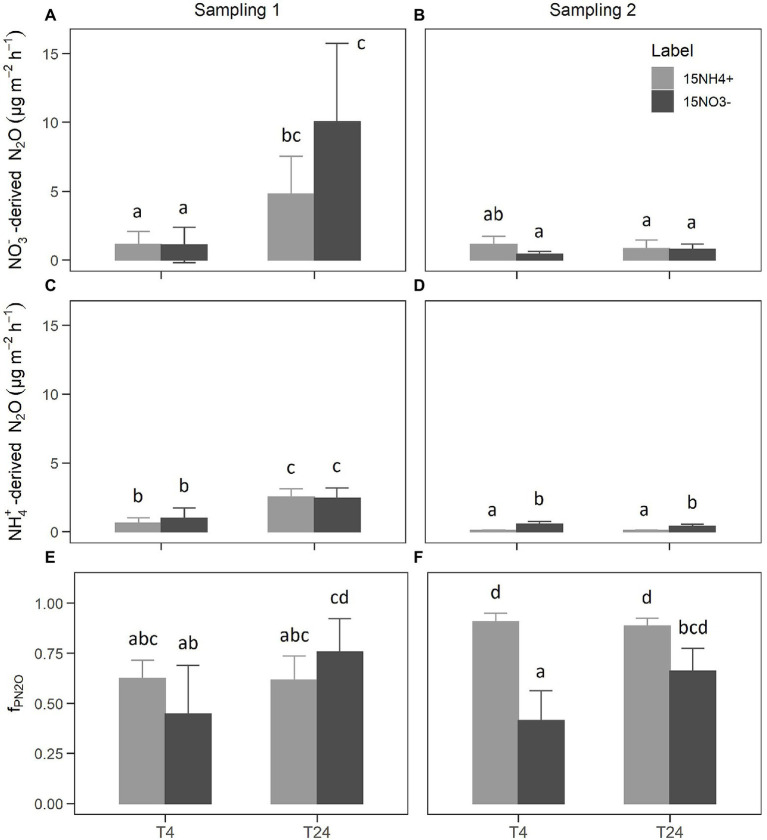
NO_3_^−^-derived N_2_O fluxes **(A,B)**, NH_4_^+^-derived N_2_O fluxes **(C,D)**, and the estimated share of NO_3_^−^-derived N_2_O fluxes [f_PN2O_; **(E,F)**]. Bars show the mean of *n* = 4 replicates and error bars the corresponding SD. Small letters indicate levels of significance for differences between label and sampling with *p* < 0.05 from linear mixed-effects models and Tukey *post-hoc* tests.

Although the same amounts of NO_3_^−^-N and NH_4_^+^-N were added in the form of NH_4_NO_3_ during each ^15^N labeling, NO_3_- concentrations were clearly higher than NH_4_+ concentrations in the nutrient solution after labeling (Table 3). This indicated that a significant amount of unlabeled nutrient solution with a high NO_3_^−^ to NH_4_^+^ ratio was still present in the rock wool substrate during ^15^N labeling. Regardless of the higher dilution of ^15^NO_3_^−^ label (Table 3; Supplementary Figure S6), the ^15^N tracer could be detected in the emitted N_2_O independent of the applied form (^15^NH_4_^+^ or ^15^NO_3_^−^). The ^15^a_N2O_ values mirrored the ^15^N enrichments of the labeled NO_3_^−^ and NH_4_^+^ pools, with higher values in of ^15^NH_4_^+^-labeled units compared to ^15^NO_3_^−^-labeled units (Supplementary Figure S6). The label dilution was considered for calculating NO_3_^−^-derived N_2_O and NH_4_^+^-derived N_2_O. The NO_3_^−^-derived N_2_O (Figures 2A,B) reflected the N_2_O emission rates measured by GC (Table 1), with highest values found 24 h after the first labeling. There was no clear difference in NO_3_^−^-derived N_2_O between the ^15^NH_4_^+^ and ^15^NO_3_^−^ labels. In general, the NH_4_^+^-derived N_2_O values (Figures 2C,D) were lower than the NO_3_^−^-derived N_2_O values, but also followed the dynamics of N_2_O emission rates measured by GC. Notably, NH_4_^+^-derived N_2_O was higher for ^15^NO_3_^−^-labeled units compared to ^15^NH_4_^+^-labeled units during sampling 2. Consequently, the calculated average f_PN2O_ values varied from 0.4 to 0.9 between the applied label forms, sampling times, and sampling campaigns (Figures 2C,D). During both sampling campaigns, an increase of f_PN2O_ from 4 h to 24 h after labeling was present for the ^15^NO_3_^−^-labeled units, while there was no effect of sampling time for the ^15^NH_4_^+^-labeled units. The latter showed higher f_PN2O_ values during the second sampling campaign, which was also significantly higher than for the ^15^NO_3_^−^-labeled units at 4 h after labeling.

**Table 3:** Concentrations and ^15^N-enrichment of dissolved ammonium and nitrate in the nutrient solution during the two sampling campaigns, including samples taken before ^15^N labeling (T0) and 4/24 h afterward (T4/T24).

Label	Sampling	Time	Dissolved NH_4_^+^		Dissolved NO_3_^−^	
			N content (mg L^−1^)	^15^N-enrichment (atom-% ^15^N excess)	N content (mg L^−1^)	^15^N-enrichment (atom-% ^15^N excess)
^15^NH_4_^+^	S1	T0	1.6 ± 0.7	–	166 ± 12	–
		T4	36 ± 9	10.04 ± 0.04	111 ± 11	0.012 ± 0.008
		T24	33 ± 6	9.96 ± 0.06	122 ± 16	0.061 ± 0.024
	S2	T0	5.9 ± 0.7	–	258 ± 11	–
		T4	61 ± 9	6.59 ± 0.04	232 ± 14	0.0004 ± 0.0018
		T24	53 ± 12	6.53 ± 0.07	250 ± 15	0.009 ± 0.007
^15^NO_3_^−^	S1	T0	1.0 ± 0.6	–	161 ± 8	–
		T4	36 ± 8	0.025 ± 0.005	124 ± 17	3.3 ± 1.2
		T24	32 ± 9	0.033 ± 0.004	131 ± 19	2.8 ± 1.0
	S2	T0	5.8 ± 0.8	–	248 ± 8	–
		T4	59 ± 11	0.007 ± 0.001	221 ± 16	2.0 ± 0.4
		T24	50 ± 10	0.007 ± 0.001	246 ± 18	1.7 ± 0.3

Shown are mean values ± SD of *n* = 4 replicates (*n* = 3 for T4 and T24 at S2 due to spillover of labeled nutrient solution between two rows).

### 3.2. Recovery of ^15^N tracer in different pools

The natural abundance δ^15^N values from both samplings were equal (leaves) or slightly lower (NH_4_^+^, NO_3_^−^ and N_2_O) at the second sampling, indicating that no carryover of ^15^N label occurred from the first sampling. The amount of ^15^N tracer from the ^15^N-enriched NH_4_NO_3_ added during the labelings that was recovered in different pools (dissolved NH_4_^+^ and NO_3_^−^, N_2_O, plant biomass) was calculated as excess ^15^N (^15^N_exc_). At both samplings, most of the ^15^N label remained in its original form after 24 h, i.e., as dissolved NH_4_^+^ and NO_3_^−^ (Table 4). There was a notable increase of ^15^N_exc_ of dissolved NO_3_^−^ in the ^15^NH_4_^+^-labeled units, indicating the conversion of NH_4_^+^ to NO_3_^−^ by Ni (up to 2% of added label during sampling 1). On the other side, the ^15^N_exc_ of dissolved NH_4_^+^ in the ^15^NO_3_^−^-labeled units was comparably low (at maximum 0.3% of added label during sampling 1). The ^15^N_exc_ of N_2_O strongly differed between the two samplings, with up to 20 times higher values at sampling 1, reflecting the APE values of N_2_O (Supplementary Figure S7). Despite the higher dilution of ^15^N tracer in the NO_3_^−^ pool (Table 3) and the resulting lower ^15^N enrichments in the ^15^NO_3_^−^-labeled units compared to ^15^NH_4_^+^-labeled units (Supplementary Figure S6), there were no significant differences between the label types regarding the amount of ^15^N tracer found in N_2_O, as shown by the ^15^N_exc_ values (Table 4). In all cases, the ^15^N_exc_ of total plant biomass was higher than the ^15^N_exc_ of N_2_O. The highest plant ^15^N uptake was observed during the second sampling in ^15^NH_4_^+^-labeled units. Irrespective of the generally higher ^15^N-enrichment of roots (Supplementary Table S2), most ^15^N tracer was found in shoots (i.e., the sum of stem leaf biomass; Table 4), as a consequence of the biomass difference (root to shoot ratio of 0.23). Only marginal amounts of ^15^N tracer were found in tomato fruits during sampling 2. Overall, the majority of ^15^N added during labelings was recovered in the studied pools, with the calculated ^15^N recovery rates varying around 100%.

**Table 4:** Excess ^15^N (^15^N_exc_) found in different pools 24 h after labeling with ^15^NH_4_^+^ and ^15^NO_3_^−^, total recovered ^15^N and recovery rate of ^15^N tracer from the labeling.

Parameter	Sampling 1		Sampling 2		Unit
	^15^NH_4_^+^ label	^15^NO_3_^−^ label	^15^NH_4_^+^ label	^15^NO_3_^−^ label	
^15^N in NH_4_^+^	96 ± 2	0.33 ± 0.03	94 ± 13*	0.09 ± 0.01*	mg ^15^N unit^−1^
^15^N in NO_3_^−^	2.1 ± 0.6	112 ± 5.42	0.54 ± 0.34*	107 ± 5*	
^15^N in N_2_O	5.0 ± 0.8^b^	4.4 ± 2.0^b^	0.22 ± 0.17^a^	0.33 ± 0.17^a^	
^15^N in shoots	5.6 ± 4.4^a^	6.4 ± 1.9^ab^	18 ± 13^b^	3.6 ± 0.9^a^	
^15^N in roots	3.9 ± 1.7^b^	1.3 ± 0.4^a^	8.1 ± 2.1^c^	1.9 ± 0.7^ab^	
^15^N in fruits	–	–	0.79 ± 0.45	BDL	
Total plant ^15^N	9.5 ± 5.4^a^	7.6 ± 2.0^a^	26 ± 15^b^	5.5 ± 0.9^a^	
Total recovered ^15^N	112 ± 5	124 ± 4	120 ± 16	111 ± 6	
^15^N recovery rate	98 ± 4	103 ± 3	105 ± 14	93 ± 5	

*Only *n* = 3 replicates due to spillover of nutrient solution between two hydroponic units. ^a–c^Small letters indicate significant differences (*p* < 0.05) between labeling and added ^15^N tracer for all parameters except dissolved NH_4_^+^ and NO_3_^−^ (^15^N source from labeling). BDL, below detection limit. Presented are mean values ± SD of *n* = 4 replicates.

## 4. Discussion

In this study, we applied the N_2_O isotopocule and ^15^N tracing approaches to better understand the sources of N_2_O emission from hydroponic vegetable production systems, using tomato cultivation on rock wool substrate as a model. Furthermore, in our study, we determined r_N2O_ using the isotopocule mapping method (Lewicka-Szczebak et al., 2017), which had been shown to be in good agreement with the ^15^N gas flux method (Buchen et al., 2018; Lewicka-Szczebak et al., 2020). Therefore, for hydroponic systems, we determined this ratio for the first using an appropriate method.

As we hypothesized, the results from both N_2_O isotope analyses (non-labeled and ^15^N-labeled) point to bD as main source of N_2_O emissions from the hydroponic units. The scattering of the values around the reduction line of bD in the mapping approach of the N_2_O isotopocules (Figure 1) suggests that most of the N_2_O was produced by bD. Unfortunately, nD cannot be clearly separated from bD by the N_2_O isotopocule mapping approach (Lewicka-Szczebak et al., 2017), due to the overlap of endmember values (i.e., theoretical values determined from literature values of pure cultures and the isotopic composition of water and N substrates). Thus, the calculated f_bD_ could actually be a mixture of bD and nD. The same is true for the fraction of Ni in N_2_O emission (f_Ni_), which cannot be clearly separated from the fraction of fD (f_fD_) in the mapping approach. However, a mixed fraction (f_Ni/fD_ = 1 – f_bD_) can be calculated, as previously done by Buchen et al. (2018). Depending on the mapping scenario and sampling campaign, the f_Ni/fD_ values vary between 0.10 and 0.15 in our study. In consequence, the contribution of fD and/or Ni seems small under typical tomato growing conditions in rock wool hydroponics with low NH_4_^+^ supply. For better distinction of bD, we used the ^15^N tracing approach to determine the fraction of NO_3_^−^-derived N_2_O fluxes, i.e., f_PN2O_. While f_PN2O_ can principally also include contributions from fD, we assume its impact was minor as shown by the isotopocule map (Figure 2). Therefore we assume f_PN2O_ is equivalent to f_bD_ from the isotopocule mapping approach but does not include N_2_O fluxes from nD. Although the f_PN2O_ values are relatively variable (Figures 2E,F), they generally show that bD was the main source of N_2_O emissions, even under increased NH_4_^+^ supply. Hence the results from N_2_O isotope analysis and ^15^N tracing were in good accordance with each other. On the other hand, the results from the ^15^N-labeling also show that a large part of N_2_O can be formed from NH_4_^+^ (Figures 2C,D), suggesting processes other than denitrification of added NO_3_^−^ (Firestone and Davidson, 1989). Possibly, the increase of the NH_4_^+^ concentration in the nutrient solution used for ^15^N-labeling compared to the non-labeled nutrient solution could have increased Ni and the associated N_2_O formation from NH_4_^+^. This is supported by the slight ^15^N-enrichment of NO_3_^−^ found in units labeled with ^15^NH_4_^+^ (Table 4), indicating the presence of Ni. Notably, the average f_bD_ values of ~0.87 from N_2_O isotopocule analysis (Table 2) were higher than the average f_PN2O_ values of ~0.68 from ^15^N tracing (Figure 2). Assuming that microbial activities did no significantly change after adding the NH_4_^+^-rich ^15^N label, we hypothesize that the observed difference in f_bD_ and f_PN2O_ values is due to microbial processes other than Ni that are associated with the release of N_2_O from NH_4_^+^.

Besides the conversion of hydroxyl amine (NH_2_OH) to N_2_O during Ni, there are several known pathways that explain the production of N_2_O derived from NH_4_^+^, in particular nD and cND (Baggs, 2011). Wrage-Mönnig et al. (2018) argue in their review that nD can be the predominant source of N_2_O emissions under certain conditions. For example, this includes “environments with fluctuating aerobic-anaerobic conditions”, which are likely to occur in hydroponic systems with regular irrigation intervals (Schröder and Lieth, 2002). In contrast, Bakken and Frostegard (2017) fundamentally disagree with the concept of nD, based on the preferential electron flow in nitrifiers, and rather suggest that it is cND that accounts for the observations after all. In this sense, the O_2_ consumption by Ni could lead to anoxic conditions facilitating bD (Zhu et al., 2015). Additionally, a process that also needs to be taken into account is co-denitrification (coD), i.e., the formation of hybrid N_2_O and N_2_ molecules with each one N atom derived from the classical denitrification pathway (N species: nitrite, NO_2_^−^; nitric oxide, NO) and one N atom from another N species such as NH_2_OH or amino compounds (Spott et al., 2011). In our study, coD may have been stimulated by the increased NH_4_^+^ availability after adding the nutrient solutions for ^15^N labeling. This is supported by the lower ap_N2O_ values compared to the ^15^aNO_3_^−^ values found in ^15^NO_3_^−^-labeled units (Supplementary Figures S6A,B,E,F; Spott and Stange, 2007), suggesting that part of the emitted N_2_O was derived from non-labeled NH_4_^+^. Albeit the use of NH_4_^+^ in coD was found quite rarely and organic N sources are thus perceived as the main source for forming hybrid N_2_O/N_2_ molecules with NO_2_^−^-N or NO-N (Spott et al., 2011). Therefore, the combined fraction of nD and cND (f_nD/cND_) can be estimated from f_PN2O_ and f_bD_ as described by Deppe et al. (2017), i.e., by calculating the difference of f_bD_ and f_PN2O_ (f_nD/cND_ = f_bD_ – f_PN2O_). Depending on the scenario for f_bD_, the values of f_nD/cND_ vary between 0.40–0.48 at T4 and 0.09–0.24 at T24 for the ^15^NO_3_^−^-labeled units during both sampling campaigns. For the ^15^NH_4_^+^-labeled units, this comparison seems not appropriate because the estimated f_PN2O_ values were partially higher than f_bD_ values. This is probably due to the assumption used in Eq. 3, i.e., that the labeled pool (^15^NO_3_^−^ and ^15^NH_4_^+^) is the same as the active pool. In contrast, the f_PN2O_ values of ^15^NO_3_^−^-labeled units were determined *via* the non-random distribution of N_2_O isotopologues and delivered the fraction of the active labeled pool used for N_2_O production, which is not necessarily identical to the bulk NO_3_^−^ pool (Deppe et al., 2017; Zaman et al., 2021).

Notably, measured N_2_O emissions from the experimental units we used were low compared to previous studies of hydroponic systems (Daum and Schenk, 1996a; Hashida et al., 2014; Karlowsky et al., 2021), which reported emission rates that were one to two orders of magnitude higher. The low N_2_O emission rates could have been a result of unfavorable conditions for denitrifier activity, such as low organic carbon contents and/or high oxygen availability in the substrate (Morley and Baggs, 2010). The accumulation of organic carbon due to root exudation and root decay might be key to N_2_O emissions from inert substrates like rock wool, as we found in a previous study a steep increase of N_2_O emission rates after 5 months of tomato cultivation following a phase of low N_2_O emission rates (Karlowsky et al., 2021). In this study, we found an increase of DOC in the re-circulating nutrient solution from sampling 1 to sampling 2, but this was not related to higher N_2_O emissions. Here, the slightly acidic conditions (pH values <4.6; Supplementary Figure S2) during sampling 2 may have limited denitrification, considering that N emissions from denitrification typically decrease at low pH values (Daum and Schenk, 1998; Farquharson and Baldock, 2007), which is also associated with a higher r_N2O_ value (e.g., Liu et al., 2010), but this was only visible in trend (Table 2). In general, N_2_O fluxes were highly variable (Table 1), with a trend to higher emissions from planted rock wool slabs compared to unplanted rock wool slabs, especially during sampling 1. Thus, our findings indicate that considerable N_2_O emissions may also occur from re-circulated nutrient solution, e.g., in collection and storage tanks or bio-filtration/disinfection units. Although it is unclear to which extent the rock wool matrix with its high pore space volumes (Dannehl et al., 2015) and a large surface area for microbial biofilms (Brand and Wohanka, 2001) might have promoted N_2_O emissions from the re-circulated nutrient solution.

In addition to the above-discussed findings, we performed a ^15^N mass balance to check the plausibility of r_N2O_ and the calculated N_2_O and N_2_ emissions from the mapping approach, and to gain more insights into N dynamics in the hydroponic units. Unfortunately, the proportion of applied ^15^N label recovered as N_2_O strongly varied between the two samplings, which can be attributed to temporal fluctuations resulting in a peak of N_2_O emission rates at 24 h after labeling during sampling 1. This peak probably led to an overestimation of cumulative N_2_O fluxes, especially considering that N_2_O emission rates are typically lower during nighttime when no fertigation is done (Daum and Schenk, 1998; Yoshihara et al., 2016; Karlowsky et al., 2021). Due to highly variable and generally very moderate N_2_O emissions as well as the high variability of ^15^N excess in plant material, the ^15^N mass balance in our case proved to be too uncertain to validate the calculated gas fluxes from the isotopocule mapping approach. In general, the results of the ^15^N mass balance reflect the findings from the ^15^N tracing approach and show in addition that the majority of ^15^N tracer applied to the hydroponic units was recovered in the nutrient solution, plant biomass, and N_2_O emissions after 24 h. However, since only short-term N dynamics are included in the ^15^N mass balance, N use efficiency cannot be calculated with these data.

## 5. Conclusion

The findings of our study clearly show that bD was the major source of N_2_O emissions from hydroponic tomato cultivation on rock wool substrate, and that up to 90% of initially produced N_2_O was reduced to N_2_ before gas emission. The combined results of N_2_O isotopocule analysis and ^15^N tracing suggest that other microbial processes related to N_2_O formation from NH_4_^+^ (i.e., Ni, nD, and cND) play only a moderate role. However, with the methods used, it was not possible to determine the individual contribution of each of these processes to the observed N_2_O emissions. Furthermore, the involvement of fD and coD remains unclear, but seems less likely since organic matter is supplied only by plant roots in the rock wool substrate. Therefore, future studies are needed to better distinguish N_2_O sources other than bD, possibly combing isotopic approaches with molecular genetic methods such as functional gene analysis. As we also found N_2_O emissions from root-less rock wool substrate, potential N_2_O emissions from drained nutrient solution should be further researched. Ultimately, on the basis of our study, measures to reduce denitrifier activity appear to be the most promising option to mitigate N_2_O emissions and N losses from hydroponic cultivation.

## Data availability statement

The raw data supporting the conclusions of this article will be made available by the authors, without undue reservation.

## Author contributions

SK: conceptualization, investigation, formal analysis, and writing—original draft. CB-T: investigation, formal analysis, and writing—original draft. LO: investigation and formal analysis. DS: conceptualization and methodology. RW: methodology and writing—review and editing. All authors contributed to the article and approved the submitted and revised version.

## Funding

This project is supported by the Federal Ministry of Food and Agriculture (BMEL) based on the decision of the Parliament of the Federal Republic of Germany *via* the Federal Office for Agriculture and Food (BLE) under the innovation support program (funding code 281B204116 for project “HydroN2O”).

## Conflict of interest

The authors declare that the research was conducted in the absence of any commercial or financial relationships that could be construed as a potential conflict of interest.

## Publisher’s note

All claims expressed in this article are solely those of the authors and do not necessarily represent those of their affiliated organizations, or those of the publisher, the editors and the reviewers. Any product that may be evaluated in this article, or claim that may be made by its manufacturer, is not guaranteed or endorsed by the publisher.
