# Supplementary material for: Sources of nitrous oxide emissions from hydroponic tomato cultivation: Evidence from stable isotope analyses
Source: Front Microbiol. 2023 Jan 4;13:1080847. doi: 10.3389/fmicb.2022.1080847 (PMC9845576; doi:10.3389/fmicb.2022.1080847)
Supplement: Supplementary file 1 [file Data_Sheet_1.docx]

Supplementary Material – ^15^N labeling in hydroponics

# Supplementary Figures


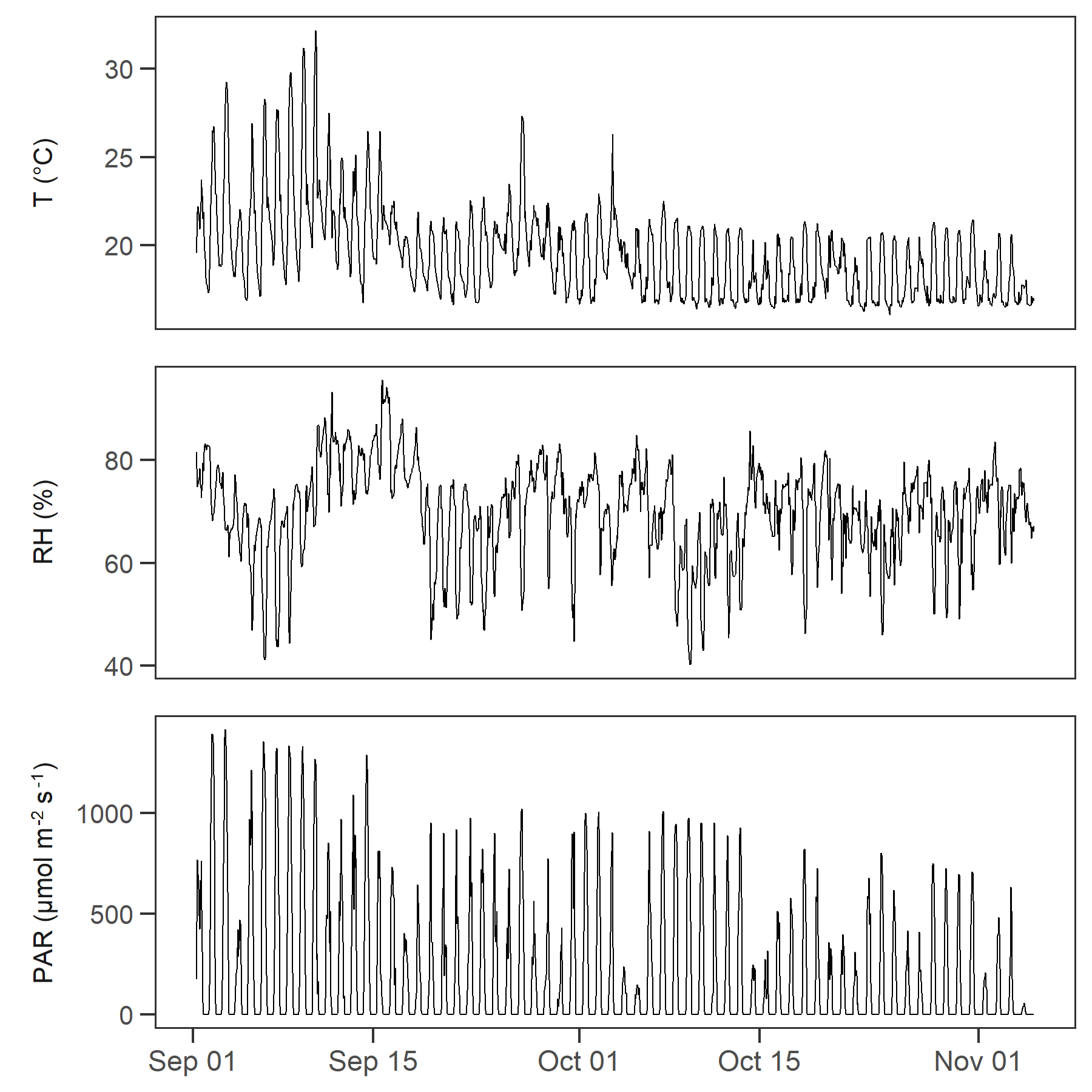


**Figure S1.** Temperature and relative humidity at 1 m height in cabin no. 5, and roof top PAR. Shown are hourly average data from 12 min measurement intervals.

**
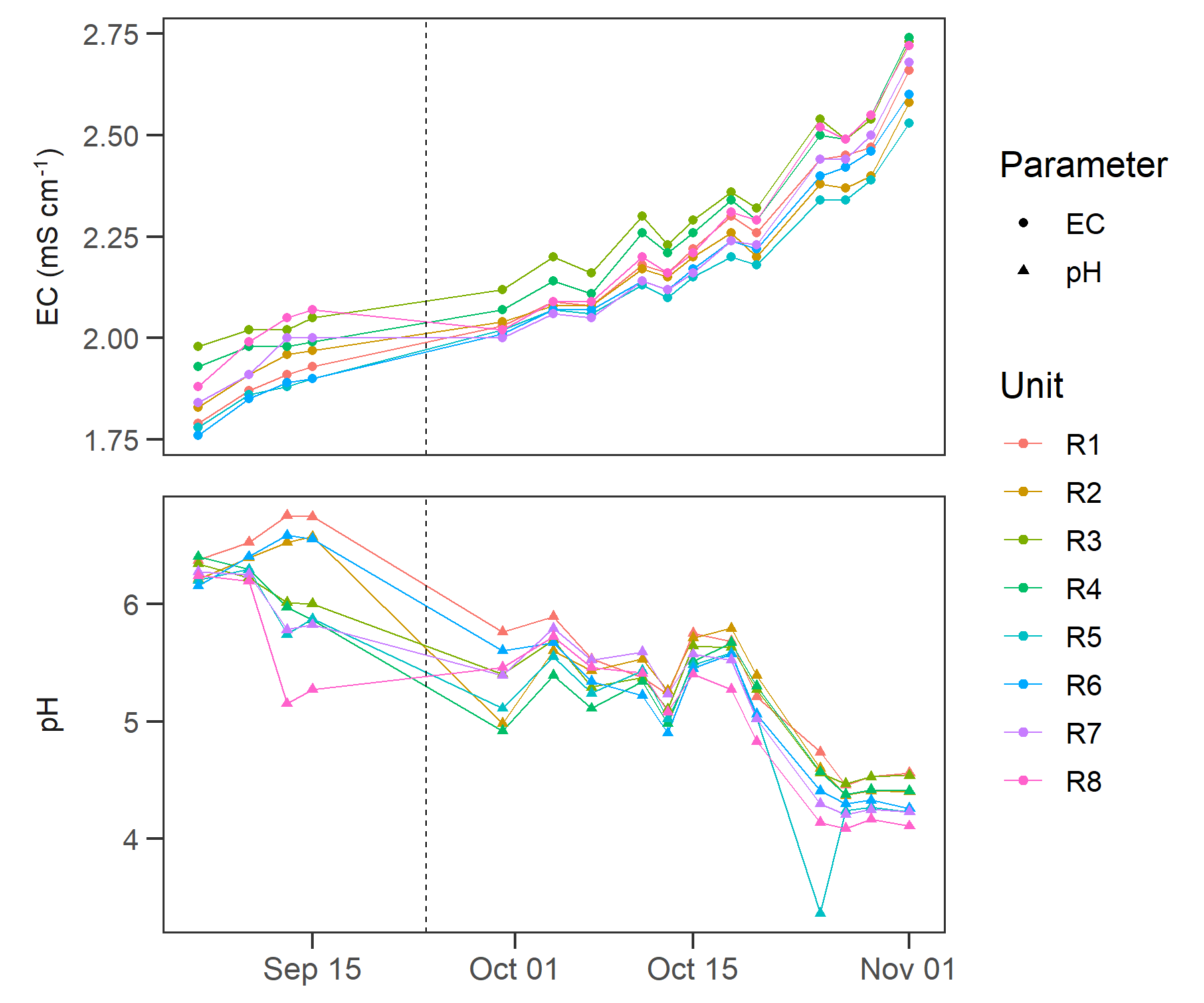
**

**Figure S2.** pH and EC values measured in nutrient solution storage tanks during tomato cultivation. Dashed lines indicate the exchange of plants after the first sampling campaign on 24^th^ September 2021.


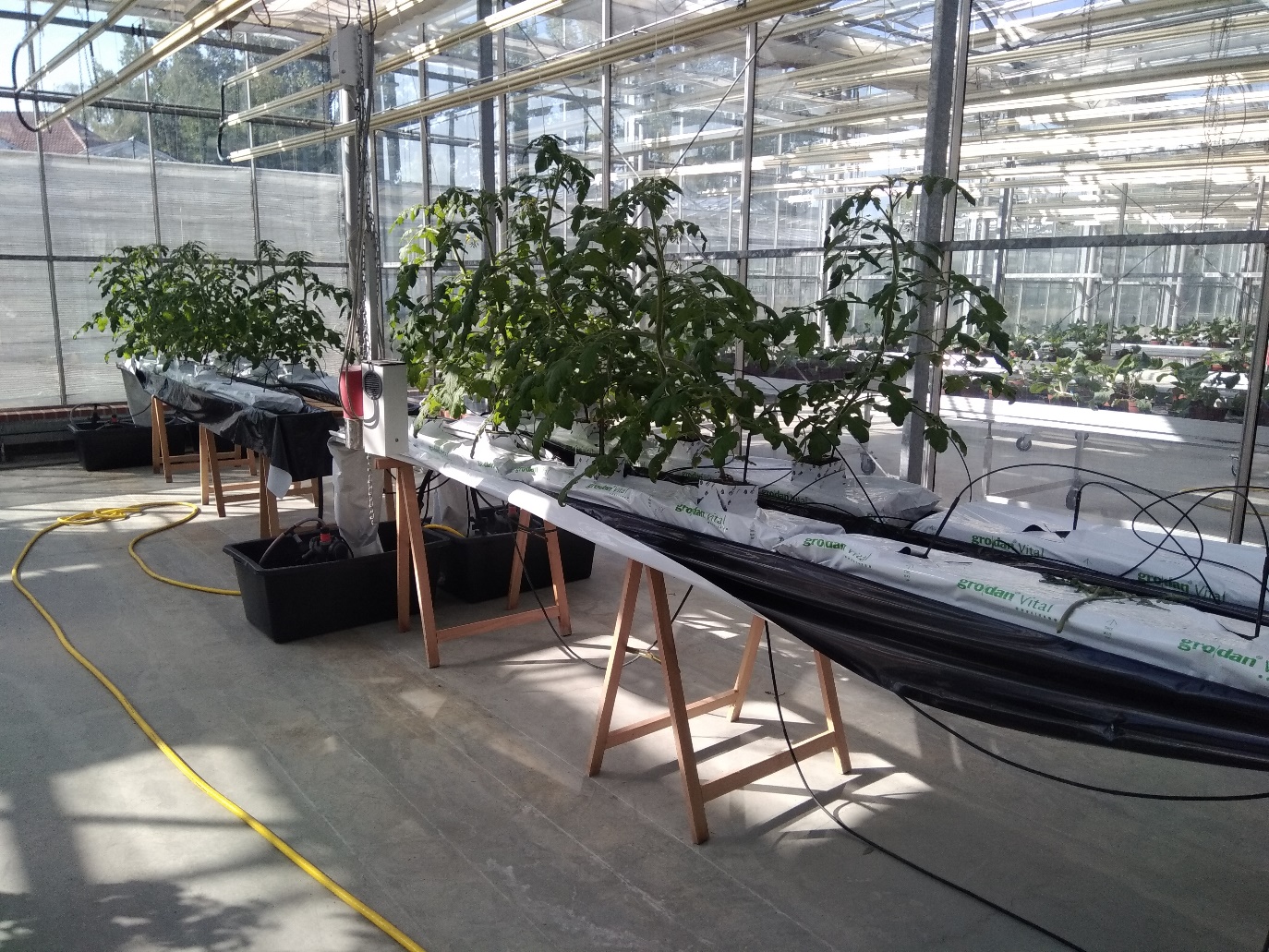


**Figure S3.** Experimental hydroponic units used for the first sampling and ^15^N labeling campaign.


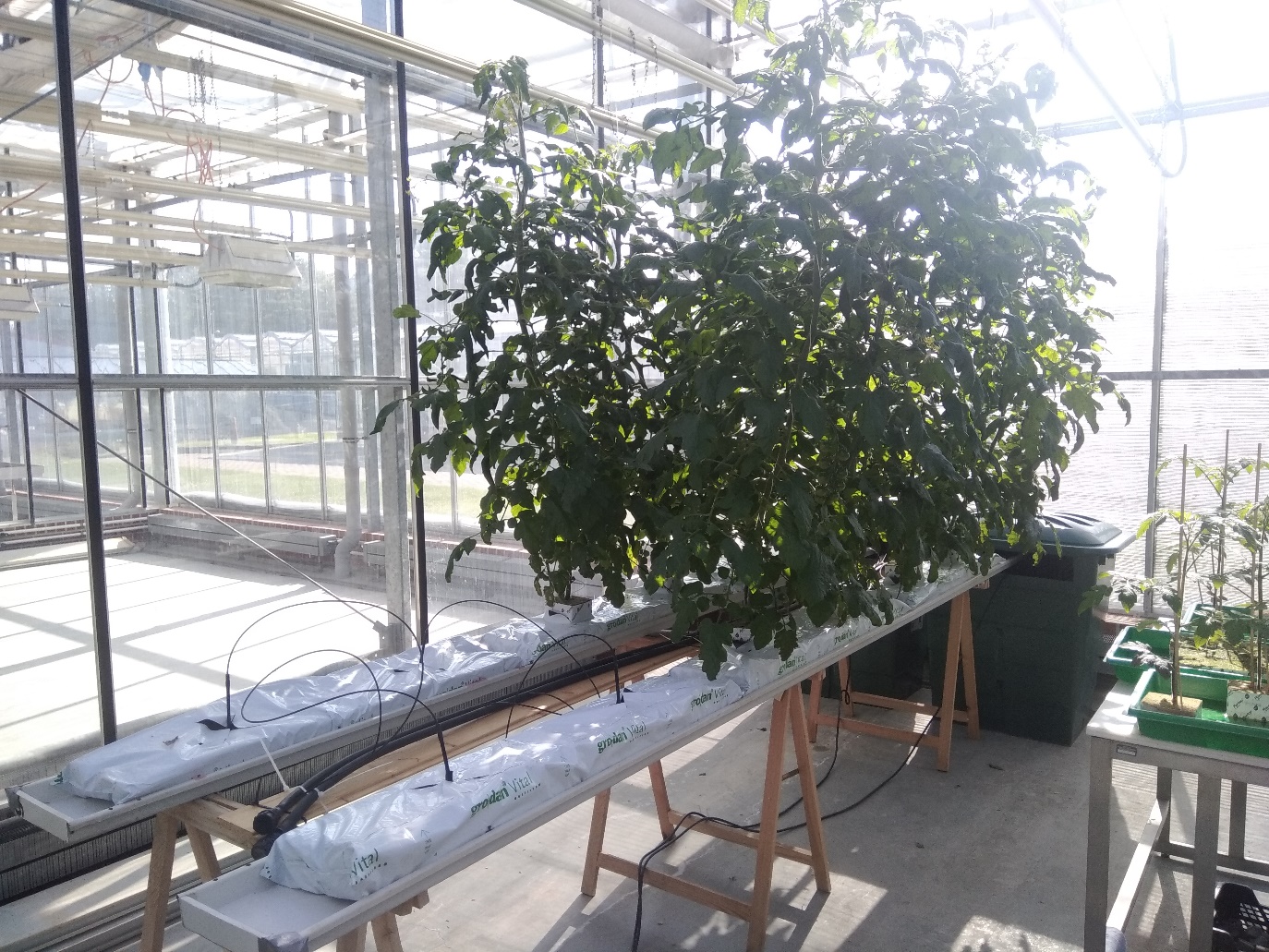


**Figure S4.** Experimental hydroponic units used for the second sampling and ^15^N labeling campaign.


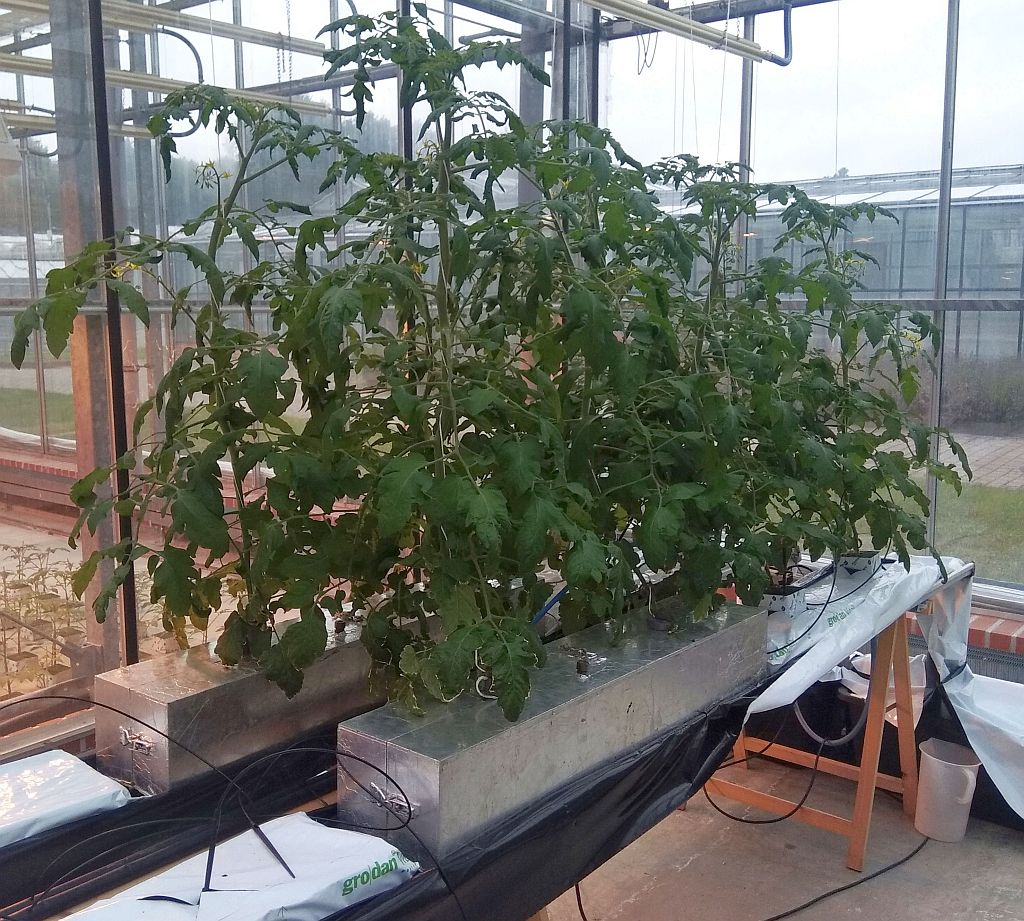


**Figure S5.** Closed chambers for gas flux measurements fitted around planted rock wool slabs during the first sampling campaign.


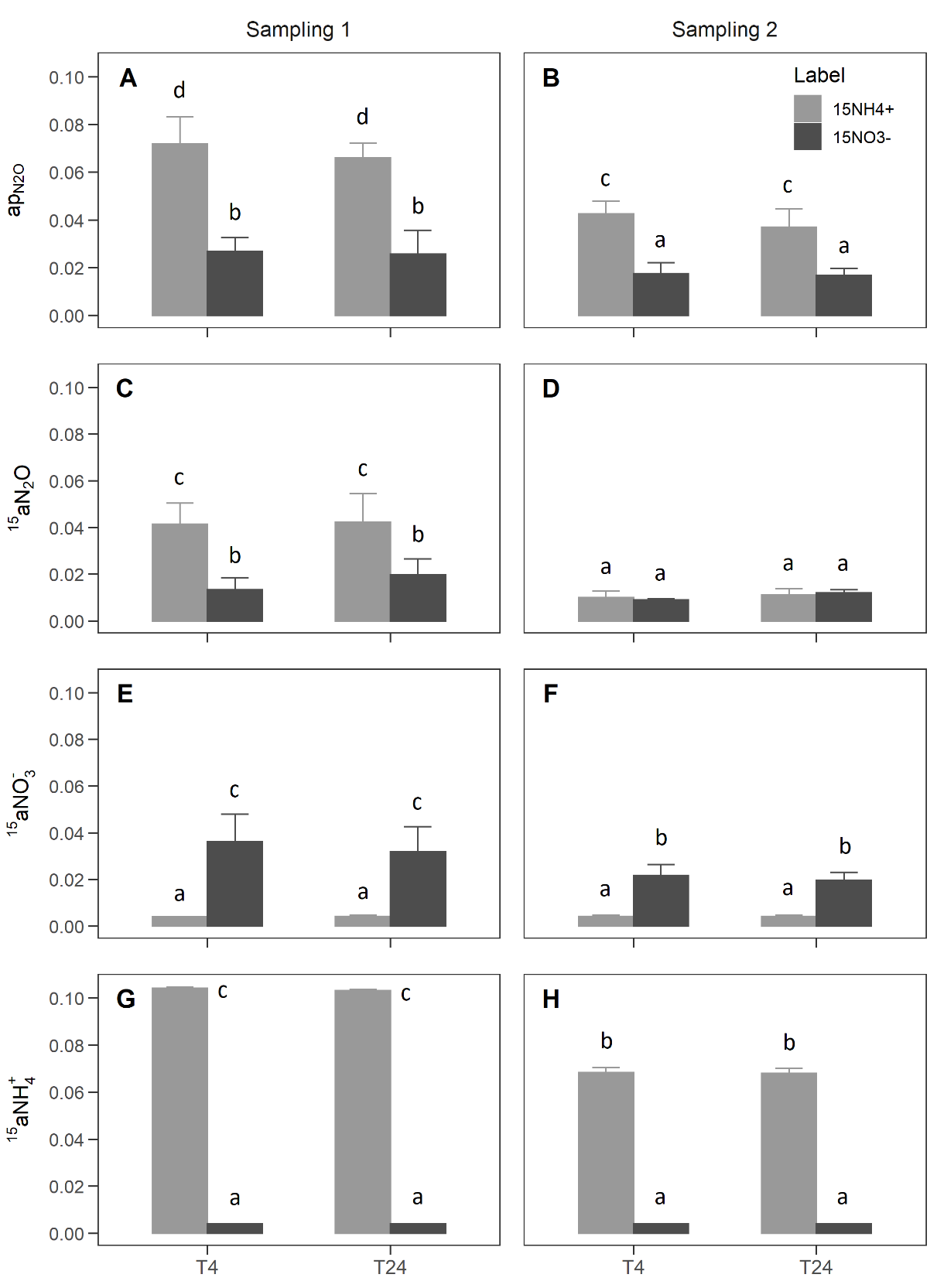


**Figure S6.** Comparison of the ^15^N enrichments (^15^N atom fractions) of the active pool for N_2_O production (ap_N2O_; A-B), emitted N_2_O (^15^aN_2_O; C-D), nitrate in the drained nutrient solution (^15^aNO_3_^-^; E-F), and ammonium in the drained nutrient solution (^15^aNH_4_^+^; G-H) during both samplings. Bars show the mean of *n* = 4 replicates and error bars the corresponding SD. Small letters indicate levels of significance for differences between label and sampling with *P* < 0.05 from linear mixed-effects models and Tukey post-hoc tests.


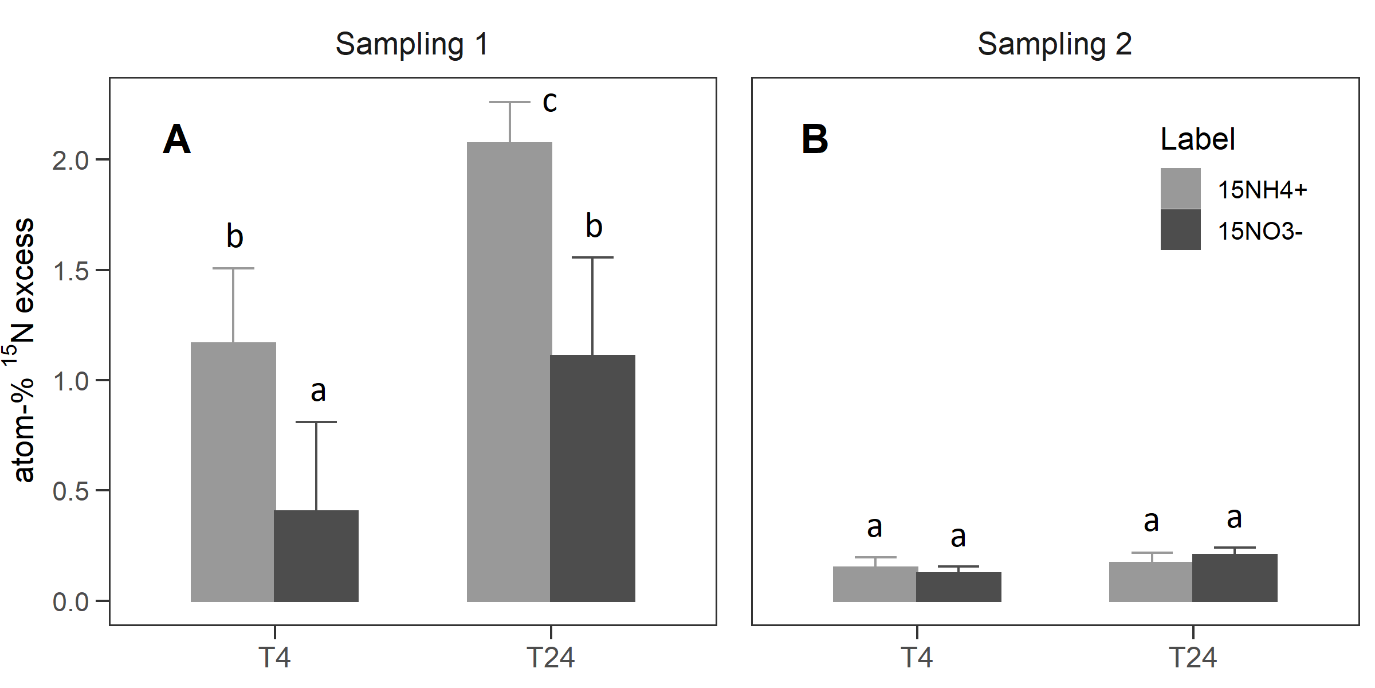


**Figure S7.** Enrichment of emitted N_2_O with ^15^N tracer (atom-% ^15^N excess) at the first sampling (A) and the second sampling (B), measured 4 h (T4) and 24 h (T24) after the labeling with ^15^N-enriched ammonium (^15^NH_4_^+^) or nitrate (^15^NO_3_^-^). Bars represent the mean of *n* = 4 replicates and error bars the corresponding SD. Small letters indicate levels of significance with *P* < 0.05 from linear mixed-effects models and a Tukey post-hoc test.

# Supplementary Tables

**Table S1.** Composition of the nutrient solutions (SOLs) used for growing tomato plants prior to labeling (starter SOL and refill SOL) and for conducting the ^15^N labeling (labeling SOL).

| Nutrient | Concentration | | | Unit |
| --- | --- | --- | --- | --- |
|  | Starter SOL | Refill SOL | Labeling SOL |  |
| *Macronutrients* | | | | |
| NO_3_^-^-N | 319 | 157 | 73 | mg L^-1^ |
| NH_4_^+^-N | 22 | 6.5 | 73 |  |
| K^+^ | 301 | 255 | 126 |  |
| PO_4_^3-^-P | 32 | 39 | 26 |  |
| Mg^2+^ | 93 | 27 | 48 |  |
| SO_4_^2-^-S | 251 | 47 | 112 |  |
| Ca^2+^ | 391 | 110 | 166 |  |
| *Micronutrients* | | | | |
| Fe^3+^ | 1380 | 840 | 1380 | µg L^-1^ |
| Mn^2+^ | 276 | 549 | 293 |  |
| Zn^2+^ | 162 | 162 | 101 |  |
| BO_3_^3-^-B | 542 | 217 | 540 |  |
| Cu^2+^ | 45 | 45 | 48 |  |
| MoO_3_-Mo | 47 | 47 | 48 |  |
| Na^+^ | 24 | 24 | 1.4 |  |
| Cl^-^ | 36 | 36 | 279 |  |

**Table S2.** Total N content (N_t_) and ^15^N-enrichment of plant biomass samples including samples taking before ^15^N labeling (T0) and 4/24 hours afterwards (T4/T24). Leaf samples were taken at all three time points, while samples from other plant parts (stem, roots and fruits) were only taken 24 hours after each labeling. Shown are mean values ± SD of *n* = 4 replicate.

| Label | Sampling | Time | N_t_ (mg g^-1^) | | | ^15^N-enrichment (atom-% ^15^N excess) | | |
| --- | --- | --- | --- | --- | --- | --- | --- | --- |
| *Leaves* | | | | | | | | |
| ^15^NH_4_^+^ | S1 | T0 | 58 | ± | 4 | - | | |
|  |  | T4 | 53 | ± | 3 | 0.027 | ± | 0.019 |
|  |  | T24 | 52 | ± | 4 | 0.053 | ± | 0.007 |
|  | S2 | T0 | 46 | ± | 4 | - | | |
|  |  | T4 | 43 | ± | 2 | BDL | | |
|  |  | T24 | 50 | ± | 1 | 0.003 | ± | 0.002 |
| ^15^NO_3_^-^ | S1 | T0 | 56 | ± | 4 | - | | |
|  |  | T4 | 54 | ± | 2 | 0.058 | ± | 0.024 |
|  |  | T24 | 53 | ± | 2 | 0.121 | ± | 0.041 |
|  | S2 | T0 | 47 | ± | 4 | - | | |
|  |  | T4 | 45 | ± | 1 | 0.005 | ± | 0.002 |
|  |  | T24 | 50 | ± | 4 | 0.012 | ± | 0.004 |
| *Stem* | | | | | | | | |
| ^15^NH_4_^+^ | S1 | T24 | 33 | ± | 2 | 0.259 | ± | 0.055 |
|  | S2 | T24 | 21 | ± | 1 | 0.146 | ± | 0.011 |
| ^15^NO_3_^-^ | S1 | T24 | 35 | ± | 2 | 0.073 | ± | 0.034 |
|  | S2 | T24 | 24 | ± | 2 | 0.020 | ± | 0.004 |
| *Roots* | | | | | | | | |
| ^15^NH_4_^+^ | S1 | T24 | 22 | ± | 2 | 0.512 | ± | 0.119 |
|  | S2 | T24 | 41 | ± | 1 | 0.210 | ± | 0.034 |
| ^15^NO_3_^-^ | S1 | T24 | 30 | ± | 3 | 0.141 | ± | 0.027 |
|  | S2 | T24 | 39 | ± | 17 | 0.057 | ± | 0.006 |
| *Fruits* | | | | | | | | |
| ^15^NH_4_^+^ | S2 | T24 | 24 | ± | 4 | 0.008 | ± | 0.003 |
| ^15^NO_3_^-^ | S2 | T24 | 24 | ± | 1 | BDL | | |

BDL, below detection limit
